# Toxicity of herbicides to the marine microalgae *Tisochrysis lutea* and *Tetraselmis* sp.

**DOI:** 10.1038/s41598-024-51401-3

**Published:** 2024-01-19

**Authors:** Florita Flores, Laura S. Stapp, Joost van Dam, Rebecca Fisher, Sarit Kaserzon, Andrew P. Negri

**Affiliations:** 1 Australian Institute of Marine Science, PMB No. 3, Townsville MC, Townsville, QLD 4810 Australia; 2 AIMS@JCU Division of Research and Innovation, Townsville, QLD 4810 Australia; 3 Australian Institute of Marine Science, Casuarina, NT 0811 Australia; 4 Indian Ocean Marine Research Centre, Australian Institute of Marine Science, University of Western Australia, Crawley, WA 6009 Australia; 5 Queensland Alliance for Environmental Health Sciences (QAEHS), The University of Queensland, Woolloongabba, QLD 4102 Australia

**Keywords:** Conservation biology, Marine biology, Ecology, Environmental sciences

## Abstract

Pesticides are ubiquitous in the catchments of the Great Barrier Reef (GBR) and regularly discharge into the nearshore waters. Effective management of pesticides requires suitable water quality guideline values (WQGVs), and further ecotoxicological data for many pesticides are needed to improve the reliability of environmental risk assessments. To help address this issue, toxicity thresholds were determined to two species of tropical marine microalgae *Tisochrysis lutea* and *Tetraselmis* sp. for a suite of herbicides detected in the GBR. Photosystem II (PSII) herbicides significantly reduced growth with no effect concentration (NEC) and 10% effect concentration (EC10) values spanning two orders of magnitude from 0.60 µg L^−1^ for diuron to 60 µg L^−1^ for simazine across both species. However, growth was insensitive to the non-PSII herbicides. The NEC/EC10 thresholds for most herbicide-microalgae combinations were greater than recent WQGVs intended to protect 99% of species (PC99); however, metribuzin was toxic to *T. lutea* at concentrations lower than the current PC99 value, which may have to be revisited. The toxicity thresholds for alternative herbicides derived here further inform the development of national and GBR-specific WQGVs, but more toxicity data is needed to develop WQGVs for the > 50 additional pesticides detected in catchments of the GBR.

## Introduction

Globally, around two million tonnes of pesticides are applied on an annual basis to agricultural crops in order to enhance crop yield and to ensure food security for an ever-growing human population^1^, with Australia using over 60,000 tonnes of pesticides in 2020^2^. Pesticides, including herbicides, insecticides, and fungicides are designed to control pest species; however, their unintentional contamination of aquatic systems has the potential to harm non-target species, potentially leading to deleterious ecological effects^1,3^. Previous research has shown that herbicide pollution, through agricultural runoff, is a potential threat to the health of the world’s largest tropical reef system, the Great Barrier Reef (GBR)^4,5^. Thirty-five rivers discharge into the GBR with large parts of these catchments downstream of areas with extensive agricultural activities where pesticides are heavily used (e.g. beef cattle grazing and sugarcane cultivation)^6,7^. This results in the year-round detection of pesticides in the Great Barrier Reef catchment area (GBRCA) with the highest concentrations usually detected in the wet season following heavy rain falls and associated runoff^8,9^. Recently, pesticides (mostly herbicides) were detected in 99.8% of over 2600 water samples collected over a five-year period from 15 waterways that discharge into the GBR lagoon^9^.

Established in 2005, the GBR Marine Monitoring Program (MMP) surveys and monitors marine water quality of the GBR and contributes data to assess risks as part of the Reef 2050 Water Quality Improvement Plan^10^. Initially, MMP pesticide monitoring focused on Photosystem II (PSII) herbicides^11^, which are extensively applied in agriculture along the Queensland coast^12^. More recently, MMP monitored 15 PSII herbicides (including breakdown products) in passive samplers at 11 fixed marine sites over the 12-month monitoring period, with herbicides diuron, atrazine and hexazinone being the most frequently detected at maximum concentrations of 250, 176 and 58 ng L^−1^, respectively^13^. A recent study reported significant increases in the concentrations of the five priority PSII herbicides (ametryn, atrazine, diuron, hexazinone, tebuthiuron) at some inshore GBR sites over the 14 years of MMP monitoring^14^. In a simulation exercise coupling end-of-system concentrations with a 3D hydrodynamic model across the entire GBR lagoon, it was indicated that diuron concentrations often exceeded 75 ng L^−1^ over 1000 km^2^ of GBR marine ecosystems during flood events^15^. The high usage of PSII herbicides, in conjunction with a high persistence in seawater, (i.e. half-lives > 100 days in seawater; Mercurio et al.^16^) illustrates why this class of herbicide represents the most frequently detected pesticides in monitored waterways^13^.

PSII herbicides inhibit photosynthesis by binding to the QB-binding site of PSII which interrupts the electron transport chain. This results in photooxidative stress and disruption of ATP synthesis, and ultimately leads to plant death^17^. The PSII complex is highly conserved in oxygenic photosynthetic organisms; thus, PSII herbicides can have negative effects on a wide range of non-target marine phototrophs. For example, PSII herbicides have been shown to inhibit photosynthesis in seagrass^18–20^, coral^21,22^, symbiotic foraminifera^23^, crustose coralline algae^24^, and growth in microalgae^25–28^ and in free living coral symbionts (Symbiodiniaceae)^29^. Due to potential risk of negative effects of PSII herbicides to key reef organisms, legislation^10,30^ and a voluntary shift in best farming practices has led to the substitution from the priority PSII herbicides (ametryn, atrazine, diuron, hexazinone, tebuthiuron) to alternative herbicides and triggered the expansion of the MMP to monitor over 40 pesticides in recent years^13,31,32^. Alternative PSII herbicides detected in inshore waters of the GBR include bromacil, metribuzin, simazine, propazine and prometryn, as well as a range of non-PSII herbicides^13,31,33^. The latter include synthetic auxins, such as 2,4‐dichlorophenoxyacetic acid (2,4-D), 2-methyl-4-chlorophenoxyacetic acid (MCPA) and fluroxypyr which mimic the plant growth hormone, resulting in abnormal plant growth, senescence, and plant death in dicots^34^. Other non-PSII herbicides detected include haloxyfop, a post-emergence herbicide, which specifically inhibits acetyl-coA carboxylase (ACCase), an enzyme critical in fatty acid synthesis^35^, and imazapic, a broad-spectrum herbicide that inhibits the acetohydroxyacid synthase enzyme (AHAS), which catalyses the first step in the synthesis of branched-chain amino acids such as valine, leucine and isoleucine^36^.

To assess environmental risks that herbicides pose to the GBR, detected herbicide concentrations have been compared against national WQGVs^37^, or more recently to merged default guideline values (DGVs) derived specifically for assessing pesticide risk to freshwater and marine ecosystems of the GBR^38^. In Australia, the preferred method to derive WQGVs for toxicants is to collate chronic toxicity threshold data for individual species into species sensitivity distributions (SSDs)^39^. SSDs are cumulative distributions of species’ responses to a given toxicant and are used to estimate concentrations that protect a certain proportion of the species community (PCx), such as 99, 95, 90 and 80% of all species (PC99, PC95, PC90 and PC80, respectively). According to the Australian and New Zealand Guidelines for Fresh and Marine Water Quality, SSDs require toxicity data for at least five species that belong to at least four taxonomic groups^37^. However, using toxicity data from at least eight species is strongly encouraged, and data for more than 15 species is considered optimal^39^.

Currently, there are national WQGVs for only 17 pesticides^37^ of the > 80 pesticides and their transformation products detected in the GBRCA^40,41^, which includes freshwater and marine WQGVs. In addition, there are GBR-specific WQGVs for 11 pesticides^42^. However, 10 of these WQGVs are characterised as being of low reliability (due to lack of chronic toxicity thresholds data for marine species). Moreover, there are no WQGVs for most alternative herbicides detected in the GBRCA. New freshwater and marine WQGVs have been proposed for 27 pesticides detected in the GBRCA^43–45^; however, many of the proposed guideline values remain at low or even very low reliability due to lack of appropriate toxicity data for tropical marine species. The reliability of marine WQGVs for use in a pesticide risk metric (PRM) has been partially addressed by applying merged fresh/marine SSDs to derive WQGVs for 22 pesticides, including the 15 that contribute to 99% of the risk to freshwater and marine GBR ecosystems^40^. Nevertheless, WQGVs for many alternative herbicides detected in the GBR remain of low to moderate reliability and/or have been derived from toxicity datasets that include few tropical marine species^37^.

Marine microalgae are widely used for routine chronic ecotoxicological testing due to their ecological importance as primary producers, short generation time, and relative ease of culturing in the laboratory e.g.^46–48^. Using two marine microalgal species from two different phyla, the haptophyte *Tisochrysis lutea* (formerly known as *Isochrysis galbana*) and the chlorophyte *Tetraselmis* sp., this study aimed to identify toxicity thresholds for a range of PSII and non-PSII herbicides to improve WQGVs for tropical marine environments. Specifically, the no-effect concentration (NEC) and the effect concentrations at 10% (EC10) and 50% (EC50) of population growth inhibition were derived for eleven and eight herbicides for *T. lutea* and *Tetraselmis* sp., respectively. Herbicides tested here were chosen according to data gaps identified in consultation with the Queensland Department of Environment and Science and included six PSII herbicides (simazine, tebuthiuron, bromacil, metribuzin, propazine and diuron as the reference herbicide^26,27,29^) and five non-PSII herbicides, the ACCase inhibitor haloxyfop-p-methyl, the acetohydroxyacid synthase inhibitor imazapic, and three auxin mimics 2,4-D, MCPA and fluroxypyr.

## Results

### Assay performance

Water quality parameters across all assays were within acceptable test limits^49^ (Table 1). Notably, the range of dissolved oxygen levels was greater likely due to measurements performed later in the day after microalgae were exposed to an extended period of light. More detailed information of water quality parameters for each treatment can be found in the online Supplementary Tables 1 and 2.

**Table 1:** Measured physico-chemical parameters, including light intensity, temperature, dissolved oxygen, pH, and salinity for all toxicity assays for *Tisochrysis lutea* (n = 30) and *Tetraselmis* sp. (n = 16).

Parameter	Range (min–max)	
	*T. lutea*	*Tetraselmis* sp.
Light intensity (µmol photons m^−2^ s^−1^; 12 h light:12 h dark)	80–100	80–100
Temperature (°C)	27–30	25–29
Dissolved oxygen (DO, mg L^−1^)	7.6–13.4	8.2–12.8
pH (units)	7.9–9.0	8.1–8.7
Salinity (PSU)	28–35	32–35

Analyses of herbicide concentrations did not detect herbicide contamination in any of the seawater controls. The average change in herbicide concentrations in test solutions between start (0 h) and end of test (72 h) measurements were within 12% for all herbicides, except for bromacil and haloxyfop, where a loss of 30% and 50% was detected, respectively. All nominal and measured concentrations of the herbicides tested can be found in the online Supplementary Tables 1 and 2.

Specific growth rates for all control samples were ≥ 0.92 day^−1^ with a coefficient of variation (CV) between control replicates ≤ 10% indicating test acceptability^49^. The control growth rate of *T. lutea* was 1.42 ± 0.23 day^−1^ (mean ± SD) with a CV of 3 ± 2% between replicates, whereas control growth rate of *Tetraselmis* sp. was 1.02 ± 0.066 day^−1^ with a mean CV of 2 ± 2% between replicates. There was no difference (p > 0.05) in SGR between the seawater controls and controls containing acetone and DMSO.

### Toxicity of PSII herbicides

**Figure 1:**
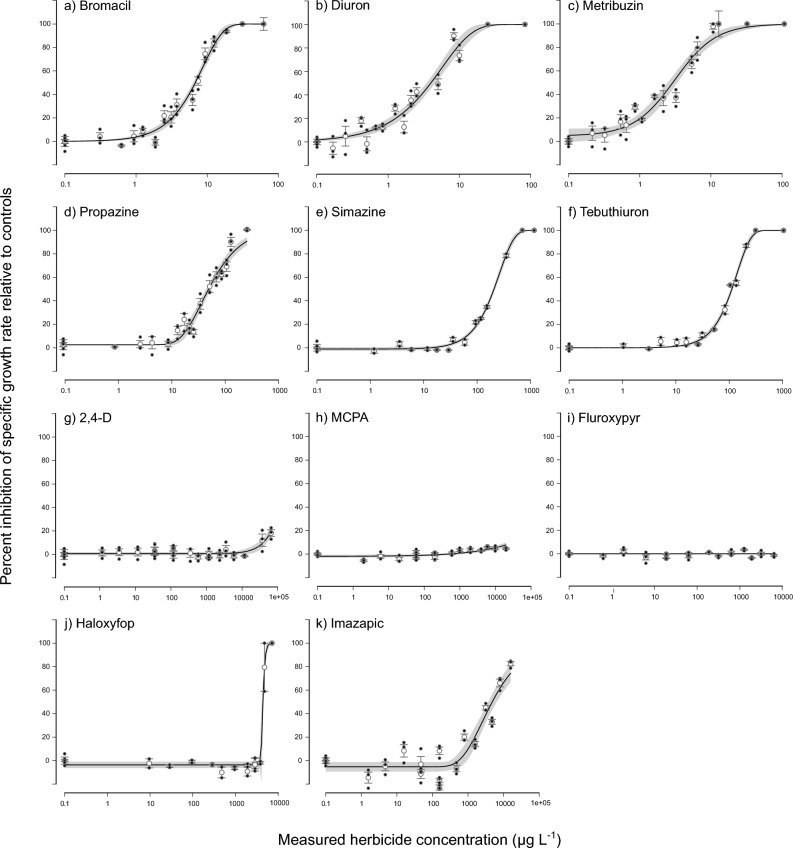
Concentration–response curves for ECx derivation. Concentration–response curves for *Tisochrysis lutea* showing the relative percent inhibition of 72 h specific growth rate (open white circles, mean ± SE) following herbicide exposure to: (**a**) bromacil, (**b**) diuron, (**c**) metribuzin, (**d**) propazine, (**e**) simazine, (**f**) tebuthiuron, (**g**) 2,4-D, (**h**) MCPA, (**i**) fluroxypyr, (**j**) haloxyfop and (**k**) imazapic. Closed black circles represent individual treatment replicates. The solid black line is the fitted regression model and the shaded areas represent the model’s 95% confidence limits. Best-fitting models (based on Akaike Information Criterion) were Weibull type II 3-parameter (bromacil, diuron, tebuthiuron), 4-parameter log-logistic (2,4-D, metribuzin), Weibull type I 3-parameter (propazine), Weibull type I 4-parameter (haloxyfop, imazapic) and Weibull type II 4-parameter (MCPA, simazine). All concentrations are reported in µg L^−1^. Note the dissimilar scaling on the horizontal axis.

**Figure 2:**
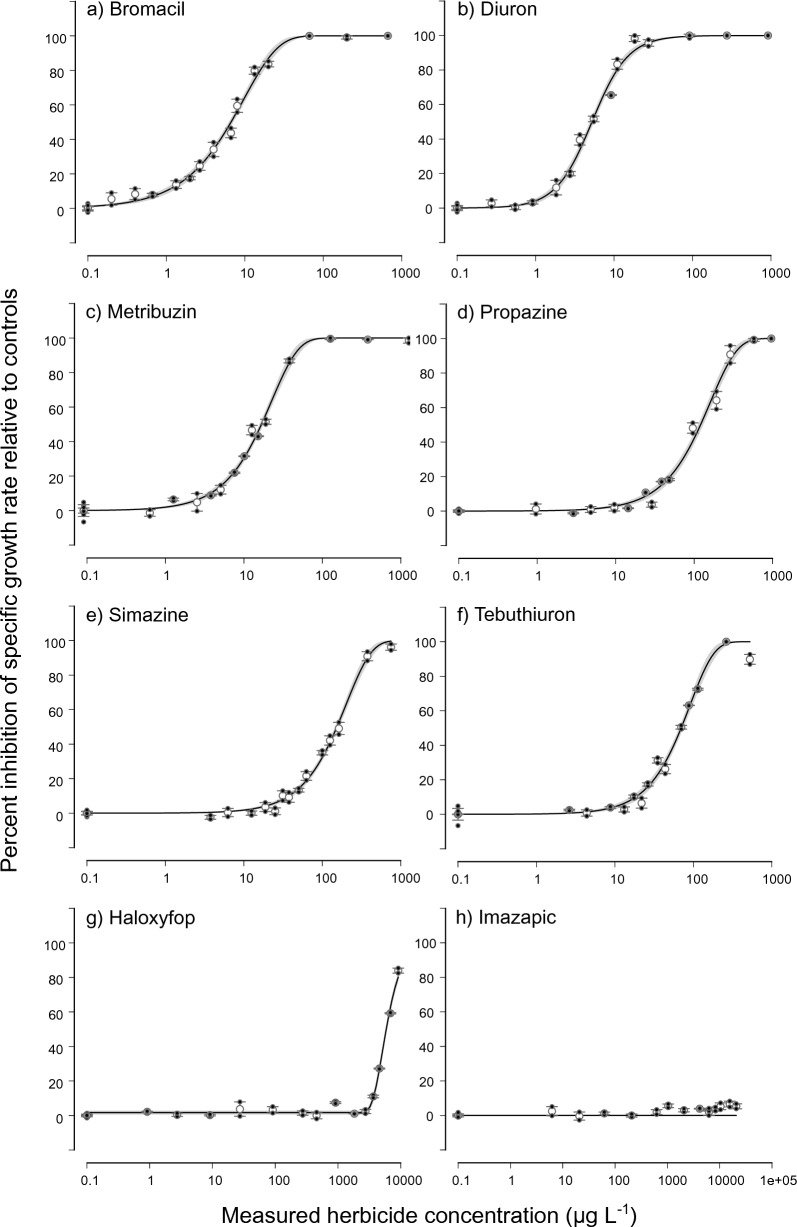
Concentration–response curves for ECx derivation. Concentration–response curves for *Tetraselmis* sp. showing the relative percent inhibition of 72 h specific growth rate (open white circles, mean ± SE) following herbicide exposure to (**a**) bromacil, (**b**) diuron, (**c**) metribuzin, (**d**) propazine, (**e**) simazine, (**f**) tebuthiuron, (**g**) haloxyfop, and (**h**) imazapic. Closed black circles represent individual treatment replicates. The solid black line is the fitted regression model and the shaded areas represent the model’s 95% confidence limits. Best-fitting models (based on Akaike Information Criterion) were Weibull type II 3-parameter (bromacil, metribuzin, propazine, simazine, tebuthiuron), 3-parameter log-logistic (diuron) and Weibull type I 4-parameter (haloxyfop). All concentrations are reported in µg L^−1^. Note the dissimilar scaling on the horizontal axis.

The inhibition of SGR of *T. lutea* and *Tetraselmis* sp. increased with increasing herbicide concentrations (Figs. 1, 2). Out of the suite of tested herbicides, PSII herbicides exhibited the highest toxicity to growth in both algal species with EC50 values of 3.1–206 µg L^−1^ for *T. lutea* and 5.2–154 µg L^−1^ for *Tetraselmis* sp. (Tables 2, 3). *T. lutea* and *Tetraselmis* sp. showed similar sensitivities to diuron and bromacil exhibiting similar EC50 toxicity thresholds (Tables 2, 3). The order of toxicity of *T. lutea* to PSII herbicides based on EC50 values was: metribuzin > diuron > bromacil > propazine > tebuthiuron > simazine (Table 2), while for *Tetraselmis* sp. diuron was most toxic and simazine remained the least toxic (Table 3). The EC50 values for all tested PSII herbicides (including the reference herbicide diuron) fell within the range of estimated EC50 values from previous research except for bromacil, with EC50 values of 6.8 and 6.7 µg L^−1^ for *T. lutea* and *Tetraselmis* sp., respectively, compared to 19–28 µg L^−1^ EC50 values for other microalgae (Table 4).

**Table 2:** Toxicity estimates for the inhibition of 11 herbicides on the specific growth rate (SGR) of *Tisochrysis lutea*.

Mode of action	Herbicide	Class	NEC	EC10	EC50	ReP
PSII inhibitor	Bromacil	Pyrimidone	2.0 (1.6–2.4)	1.9 (1.6–2.3)	6.8 (6.3–7.3)	0.59
	Diuron	Phenylurea	0.78 (0.44–1.3)	0.60 (0.40–0.80)	4.0 (3.4–4.5)	1
	Metribuzin	Triazine	0.50 (0.29–1.2)	0.72 (0.36–1.1)	3.1 (2.5–3.8)	1.3
	Propazine	Triazine	14.4 (10.8–20.9)	18.5 (15.2–21.9)	56.5 (51.0–62.0)	0.071
	Simazine	Triazine	70.0 (55.3–80.3)	60.2 (51.9–68.4)	206 (194–218)	0.019
	Tebuthiuron	Urea	63.1 (42.5–71.5)	35.9 (30.6–41.1)	112 (106–118)	0.036
ACCase inhibitor	Haloxyfop	Pyridine	4180 (3800–4710)	4000 (3650–4350)	4380 (4160–4600)	0.00091
AHAS inhibitor	Imazapic	Imidazolinone	471 (283–861)	783 (399–1170)	4320 (3180–5460)	0.00093
Auxin mimic	2,4-D	Phenoxy-alkane	15,300 (6980–28,400)	40,700 (28,800–52,500)	172,000 (61,500–283,000)	2.3 × 10^–5^
	MCPA	Phenoxy	Unreliable NEC*	21,800 (7670–35,900)	> 20,000,000	NA
	Fluroxypyr	Aminopyridine	Unreliable NEC*	> 6300	> 6300	NA

Concentration–response curves estimating no effect concentrations can be found in Supplementary Fig. 1. The potencies for each of the herbicides were contrasted using the relative equivalent potencies in comparison to diuron (ReP = EC50_diuron_/EC50_herbicide_), which has conventionally been used as a reference herbicide for the comparison of herbicide potency^26,27,29^. NA indicates values could not be calculated. All concentrations are in µg L^−1^ (95% confidence intervals).

*Although a NEC was provided by the model (Supplementary Fig. 1), no concentration–response relationship was observed and confidence around the supplied NEC was extremely low. Therefore, the NEC was deemed unreliable and was not included here.

**Table 3:** Toxicity estimates for the inhibition of eight herbicides on the specific growth rate (SGR) of *Tetraselmis* sp. concentration–response curves estimating no effect concentrations can be found in Supplementary Fig. 2.

Mode of action	Herbicide	Chemical class	NEC	EC10	EC50	ReP
PSII inhibitor	Bromacil	Pyrimidone	1.8 (1.3–2.4)	0.99 (0.79–1.2)	6.7 (6.2–7.1)	0.78
	Diuron	Phenylurea	2.3 (2.0–2.5)	1.6 (1.4–1.9)	5.2 (4.9–5.6)	1
	Metribuzin	Triazine	6.7 (4.7–7.8)	4.1 (3.5–4.8)	18.5 (17.4–19.5)	0.28
	Propazine	Triazine	29.3 (22.2–34.5)	27.2 (22.4–32.0)	121 (111–130)	0.043
	Simazine	Triazine	37.5 (27.9–46.3)	37.6 (33.0–42.2)	154 (145–162)	0.034
	Tebuthiuron	Phenylurea	20.6 (15.7–24.6)	18.4 (15.4–21.4)	69.9 (65.5–74.4)	0.074
ACCase inhibitor	Haloxyfop	Pyridine	Unreliable NEC*	3740 (3560–3930)	5930 (5740–6110)	0.00088
AHAS inhibitor	Imazapic	Imidazolinone	Unreliable NEC*	> 20,800	> 20,800	NA

The potencies for each of the herbicides were contrasted using the relative equivalent potencies in comparison to diuron (ReP = EC50_diuron_/EC50_herbicide_), which has conventionally been used as a reference herbicide for the comparison of herbicide potency^26,27,29^. NA indicates values could not be calculated. All concentrations are in µg L^−1^ (95% confidence intervals).

*Although a NEC was provided by the model (Supplementary Fig. 2), no concentration–response relationship was observed and confidence around the supplied NEC was extremely low. Therefore, the NEC was deemed unreliable and was not included here.

**Table 4:** Herbicide toxicity values (NEC, EC10, EC50) on the growth rate of *Tisochrysis lutea*, *Tetraselmis* sp., and a selection of other marine microalgae.

Herbicide	Phylum	Species	Test duration (days)	NEC/EC10* (µg L^−1^)	EC50 (µg L^−1^)	References
Bromacil	Haptista	*Tisochrysis lutea*	3	1.94	6.80	Present study (Table 2)
	Chlorophyta	*Tetraselmis* sp.	3	0.99	6.68	Present study (Table 3)
	Cryptophyta	*Rhodomonas salina*	3	4.89	19.3	Thomas et al.^26^
	Bacillariophyta	*Skeletonema costatum*	5		25	USEPA^50^
	Dinoflagellata	*Cladocopium proliferum (formerly C. goreaui)*	14	16.6	27.7	Marzonie et al.^29^
Diuron	Haptista	*Tisochrysis lutea*	3	0.60	3.96	Present study (Table 2)
		*Tisochrysis lutea*	4		2.20	Dupraz et al.^51^
		*Tisochrysis lutea*	4		3.73	Dupraz et al.^52^
	Chlorophyta	*Tetraselmis* sp.	3	1.64	5.24	Present study (Table 3)
		*Tetraselmis suecica*	4		4.20	Dupraz et al.^52^
		*Nephroselmis pyriformis*	3	5.1	7.7	Magnusson et al.^25^
		*Dunaliella tertiolecta*	4		9.2	DeLorenzo et al.^53^
	Cryptophyta	*Rhodomonas salina*	3	1.68	6.27	Thomas et al.^26^
	Bacillariophyta	*Chaetoceros muelleri*	3	1.47	12.4	Thomas et al.^27^
		*Skeletonema marinoi*	4		10.3	Dupraz et al.^51^
		*Navicula* sp.	3	2.3	7.7	Magnusson et al.^54^
		*Thalassiosira pseudonana*	4	1.6	4.3	Bao et al.^55^
		*Skeletonema costatum*	4	3.8	5.9	Bao et al.^55^
		*Navicula forcipata*	4		27	Gatidou et al.^56^
		*Nitzschia pungens*	4		6.6	Jung et al.^57^
		*Chaetoceros gracilis*	3		36	Koutsaftis and Aoyama^58^
	Cyanobacteria	*Chroococcus minor*	7	0.44	4.7	Bao et al.^55^
		*Synechococcus* sp.	4	12	110	Bao et al.^55^
		*Synechococcus* sp.	3		0.55	Devilla et al.^59^
	Dinoflagellata	*Cladocopium proliferum*	14	2.54	4.45	Marzonie et al.^29^
	Haptophyta	*Coccolithus huxleyi*	3		2.3	Devilla et al.^59^
Metribuzin	Haptista	*Tisochrysis lutea*	3	0.50	3.11	Present study (Table 2)
	Chlorophyta	*Tetraselmis* sp.	3	4.14	18.5	Present study (Table 3)
	Cryptophyta	*Rhodomonas salina*	3	2.21	13.4	Thomas et al.^26^
	Bacillariophyta	*Skeletonema costatum*	5		88	USEPA^50^
	Dinoflagellata	*Cladocopium proliferum*	14	22.3	33.5	Marzonie et al.^29^
Propazine	Haptista	*Tisochrysis lutea*	3	14.4	56.5	Present study (Table 2)
	Chlorophyta	*Tetraselmis* sp.	3	27.2	121	Present study (Table 3)
	Cryptophyta	*Rhodomonas salina*	3	27.8	188	Thomas et al.^26^
	Bacillariophyta	*Chaetoceros muelleri*	3	12.9	98.2	Thomas et al.^27^
		*Skeletonema costatum*	5		25	USEPA^50^
	Dinoflagellata	*Cladocopium proliferum*	14	45.1	86.5	Marzonie et al.^29^
Simazine	Haptista	*Tisochrysis lutea*	3	60.2	206	Present study (Table 2)
	Chlorophyta	*Tetraselmis* sp.	3	37.5	154	Present study (Table 3)
	Cryptophyta	*Rhodomonas salina*	3	38.4	184	Thomas et al.^26^
	Bacillariophyta	*Skeletonema costatum*	5		60	USEPA^50^
		*Ceratoneis closterium*	4	310	> 1000	Hook et al.^60^
		*Phaeodactylum tricornutum*	3	100	580	Osborn and Hook^61^
	Dinoflagellata	*Cladocopium proliferum*	14	257	387	Marzonie et al.^29^
Tebuthiuron	Haptista	*Tisochrysis lutea*	3	35.9	112	Present study (Table 2)
	Chlorophyta	*Tetraselmis* sp.	3	18.4	69.9	Present study (Table 3)
	Cryptophyta	*Rhodomonas salina*	3	22.7	112	Thomas et al.^26^
	Bacillariophyta	*Chaetoceros muelleri*	3	16.0	187	Thomas et al.^27^
		*Skeletonema costatum*	5		60	USEPA^50^
	Dinoflagellata	*Cladocopium proliferum*	14	107	331	Marzonie et al.^29^
Haloxyfop	Haptista	*Tisochrysis lutea*	3	4000	4380	Present study (Table 2)
	Chlorophyta	*Tetraselmis* sp.	3	3740	5930	Present study (Table 3)
	Cryptophyta	*Rhodomonas salina*	3	> 3700	> 3700	Thomas et al.^26^
	Bacillariophyta	*Chaetoceros muelleri*	3	> 4570	> 4570	Thomas et al.^27^
	Dinoflagellata	*Cladocopium proliferum*	14	> 2980	> 2980	Marzonie et al.^29^
Imazapic	Haptista	*Tisochrysis lutea*	3	471	4320	Present study (Table 2)
	Chlorophyta	*Tetraselmis* sp.	3	> 20,800	> 20,800	Present study (Table 3)
		*Nephroselmis pyriformis*	3, 5, 10		< 1455	Magnusson^62^
	Crytophyta	*Rhodomonas salina*	3	410,000	790,000	Thomas et al.^26^
	Bacillariophyta	*Skeletonema costatum*	5		< 45	USEPA^50^
		*Navicula* sp.	3, 5, 10		< 1455	Magnusson^62^
	Dinoflagellata	*Cladocopium proliferum*	14	> 165,000	> 165,000	Marzonie et al.^29^
2,4-D	Haptista	*Tisochrysis lutea*	3	15,300	172,000	Present study (Table 2)
	Cryptophyta	*Rhodomonas salina*	3	> 279,000	> 279,000	Thomas et al.^26^
	Bacillariophyta	*Skeletenoma costatum*	5		> 2000	USEPA^50^
		*Chaetoceros calcitrans*	21		9200	His and Seaman^63^
MCPA	Haptista	*Tisochrysis lutea*	3	21,800	> 20,000,000	Present study (Table 2)
Fluroxypyr	Haptista	*Tisochrysis lutea*	3	> 6300	> 6300	Present study (Table 2)

Table adapted from Thomas et al.^26^.

*The lowest toxicity threshold of the NEC or EC10 was reported.

### Toxicity of non-PSII herbicides

For both algal species, the EC50 values of non-PSII herbicides were at least an order of magnitude higher compared to the PSII herbicides (Tables 2, 3). While the acetyl-CoA carboxylase inhibitor haloxyfop and the acetohydroxyacid synthase (AHAS) inhibitor imazapic exhibited similar EC50s of around 4300 µg L^−1^ for *T. lutea* (Table 2), the auxin mimics 2,4 D, MCPA and fluroxypyr hardly affected growth at the highest concentrations tested and EC50 concentrations could not be estimated for MCPA and fluroxypyr within the tested concentration range (Fig. 1, Table 2). Similarly, haloxyfop was > 1000 times less toxic to the growth of *Tetraselmis* sp. than the most toxic PSII herbicide diuron and the EC50 could not be estimated for imazapic within the tested concentration range (Fig. 2, Table 3). The EC50 values that could be estimated were within the same concentration range as reported in previous studies (Table 4).

## Discussion

This study extends toxicity data for a suite of PSII herbicides and non-PSII herbicides commonly detected in the Great Barrier Reef catchment area (GBRCA) for two relevant marine microalgae species, *Tisochrysis lutea* and *Tetraselmis* sp. to further inform the development of national water quality guideline values (WQGVs) and GBR-specific default guideline values (DGVs) and associated ecological risk assessments for pesticide mixtures. Consistent with previous studies, PSII herbicides were at least an order of magnitude more toxic to marine microalgae than non-PSII herbicides. The NEC/EC10 thresholds for most herbicide-microalgae combinations were greater than recent PC99 DGVs, indicating adequate protection; however, the PSII herbicide metribuzin was toxic to *T. lutea* at concentrations lower than the most recent PC99 values, which may have to be revisited. The reproducibility of control growth rates and toxicity estimates identified for these two microalgal species reinforces their suitability for routine ecotoxicity testing^46–48,64^.

### Toxicity of PSII herbicides to *T. lutea* and *Tetraselmis* sp.

Chronic exposure to the six different PSII herbicides significantly reduced growth in both test species, *T. lutea* and *Tetraselmis* sp. Despite their shared mode of action, there is a significant difference in toxicity among the PSII herbicides, with bromacil, diuron, and metribuzin displaying a much greater toxicity in both species compared to the other PSII herbicides propazine, simazine and tebuthiuron. Interestingly, the differences in toxicity do not seem to be related to the chemical class of these herbicides. For instance, diuron and tebuthiuron belong to the same phenylurea class, yet diuron was 28- and 13-fold more toxic than tebuthiuron to *T. lutea* and *Tetraselmis* sp., respectively. Such a disparity in toxicity between these two herbicides aligns with other studies whereby diuron was 6–150-fold more toxic than tebuthiuron to other marine microalgae, including *Rhodomonas salina*, *Chaetoceros muelleri*, *Cladocopium proliferum* and *Skeletonema costatum*. Marzonie et al.^29^ reported no correlation between toxicity and the octanol–water partition coefficient among these herbicides, implying that the ability to cross algal cell walls and membranes or accumulate within cells does not significantly influence their relative toxicity. Instead, factors including steric compatibility (the spatial arrangement of atoms or groups) and specific affinity of each herbicide for the QB binding site on the D1 protein in PSII are likely to be the key determinants of relative toxicity^65^. Drifts in pH during the experiments (less than 1.5 pH units) were unlikely to have affected the solubility and bioavailability of any of the herbicides (pKa values > 2 from the experimental pH range), with the potential exception of bromacil which has a pKa of 9.3. Bromacil would become more soluble as the pH increased, but any effects on toxicity are unknown.

### Toxicity of non-PSII herbicides to *T. lutea* and *Tetraselmis* sp.

*T. lutea* and *Tetraselmis* sp. were insensitive to the non-PSII herbicides tested here with either relatively high or no EC50 values estimated for all non-PSII herbicides. Auxin regulators, including 2, 4-D, MCPA and fluroxypyr, are primarily used as selective herbicides for controlling dicotyledons (i.e. broadleaves) but not most monocotyledons (i.e. rice, wheat, maize) by mimicking the action of the plant hormone auxin resulting in uncontrolled growth and eventually plant death. Though microalgae have been observed to produce phytohormones, including auxins, they are present at very low concentrations^66^, likely explaining the low sensitivity of *T. lutea* to these herbicides. Haloxyfop inhibits acetyl-CoA carboxylase (ACCase), a key enzyme involved in the biosynthesis of fatty acids. This enzyme comes in two isoforms, the prokaryotic (heteromeric) form and eukaryotic (homomeric) form. The heteromeric form of this enzyme is found in the plastids of plants and algae while the homomeric ACCase is found in the cytosol of plants and algae^67,68^. ACCase inhibitors, including haloxyfop, bind to and block the eukaryote-type homomeric ACCase enzyme^45^. Studies have found that the microalgae *Chlorella variabilis* and *T. lutea* contain the heteromeric form of ACCase^68,69^, likely explaining the lack of response of haloxyfop to *T. lutea* and *Tetraselmis* sp. Additionally, the cryptophyte *Rhodomonas salina,* diatom *Chaetoceros muelleri,* and dinoflagellate *Cladocopium proliferum* were insensitive to haloxyfop^26,27,29^ suggesting this mode of action is unlikely to have a deleterious effect on the population growth of microalgae.

Imidazolinone herbicides, such as imazapic, act by inhibiting the acetohydroxyacid synthase (AHAS), an enzyme important in the synthesis of three branched-chain aliphatic amino acids leucine, isoleucine, and valine in plants, fungi and microorganisms^70^. AHAS-inhibiting herbicides have been commercially applied to crops for several decades and since then 197 site-of-action resistance isolates have been identified in weeds^71^. Lonhienne et al.^71^ demonstrated that these mutations reduced the binding affinity of these herbicides and prohibited time-dependent accumulative inhibition. Time-dependent accumulative inhibition is the prolonged effect of the inactivation of AHAS long after the inhibitor (herbicide) has left the catalytic site. It is feasible that *T. lutea* and *Tetraselmis* sp. also possess resistance isolates explaining their lack of sensitivity to imazapic. Additionally, Thomas et al.^26^ hypothesized that the structure of imazapic may affect its bioavailability in seawater. Imazapic has a carboxylic acid group which may complex with Mg and Ca ions in seawater reducing the availability of the herbicide to surrounding organisms.

### Implications of water quality guideline development

Although > 80 pesticides and their transformation products have been detected in the GBRCA^40,41^, only 17 have national water quality guideline values, often of low reliability, due to lack of appropriate marine toxicity data^37^. Of the herbicides tested here, only bromacil, diuron, simazine, tebuthiuron, 2,4-D and MCPA have existing WQGVs, with no current WQGVs for metribuzin, propazine, haloxyfop, imazapic, and fluroxypyr. A comparison of the existing national marine WQGVs^37^, proposed marine guideline values^44,45^ and the more recent merged default guideline values^38^ against growth toxicity thresholds (NEC/EC10, the lower of the two) for *T. lutea* and *Tetraselmis* sp. is presented in Table 5. The PC99 WQGV for bromacil is inadequate to protect either of the microalgal species since the estimated toxicity thresholds for bromacil are two orders of magnitude lower than the current WQGV. However, the current PC99 WQGVs for diuron, simazine, 2,4-D, and MCPA are protective of *T. lutea* and *Tetraselmis* sp. The more recent PGVs and DGVs for all herbicides are all lower than the NEC/EC10 values for both *T. lutea* and *Tetraselmis* sp., indicating sufficient protection for these herbicide/algae species combinations except for metribuzin. The NEC (0.50 µg L^−1^) and EC10 (0.72 µg L^−1^) thresholds for metribuzin to *T. lutea* are lower than the PGV and DGV value of 2.0 µg L^−1^ (Table 5) which may have to be revisited.

**Table 5:** Comparison of water quality guideline values (WQGVs; ANZG^37^), proposed water quality guideline values (PGVs; King et al.^44^; King et al.^45^) and merged default guideline values for use in the Pesticide Risk Metric (DGVs; Warne et al.^38^) for 99% species protection against toxicity thresholds estimated for *Tisochrysis lutea* and *Tetraselmis* sp. from this study.

Herbicide	WQGV—PC99	PGV—PC99	DGV—PC99	*T. lutea*—NEC/EC10*	*Tetraselmis* sp.—NEC/EC10*
Bromacil	180	0.23	NA	1.94	0.99
Diuron	0.2	0.43	0.075	0.6	1.64
Metribuzin	NA	2.0	2.0	0.5	4.14
Propazine	NA	2.2	NA	14.4	27.2
Simazine	0.2	28	17	60.2	37.5
Tebuthiuron	0.02	4.7	4.7	35.9	18.4
Haloxyfop	NA	590	589	4000	3740
Imazapic	NA	0.049	0.049	471	> 20,800
2,4-D	140	1000	7.3	15,300	–
MCPA	1.4	1	0.0075	21,800	–
Fluroxypyr	NA	87	114	> 6300	–

The DGV—PC99 values, generated from the most up-to-date combined fresh/marine SSDs, are applied in the Pesticide Risk Metric for GBR waters^38^. All concentrations are in µg L^−1^. NA denotes no available guideline value while (–) denotes no threshold value was determined. Note that DGVs are generated using the combined freshwater and marine toxicity data.

*The lowest toxicity threshold of the NEC or EC10.

The NEC and EC10 thresholds derived for these herbicides are higher than most concentrations reported in recent MMP surveys of pesticides from month-long passive sampler deployments^13,31,32^. However, based on a simulation exercise coupling end of system diuron concentrations with a 3D hydrodynamic model, orders of magnitude higher concentrations are expected over short durations (hours to days) in marine waters of the GBR^15^, often exceeding WQGVs and the NEC/EC10 thresholds for diuron reported here. Importantly, the vast majority (> 90%) of marine samples taken as part of the MMP in the three most recent surveys comprise mixtures of more than one pesticide^13,32^, and assessments of risk should always consider the contributions of all pesticides detected, rather than exceedances of individual WQGVs separately^38^. The ‘multisubstance-potentially affected fraction’ (ms-PAF) approach has recently been implemented in MMP reporting to help address this issue^13,31,32^. The ms-PAF method assesses the cumulative ecological risk of pesticide mixtures for species assemblages by deriving the percentage of species that would be affected by each pesticide alone and combining with an additivity model to predict the joint percent affected fraction^72^. Exceedances of PC99 in nearshore waters of the GBR are more frequent when accounting for the effect of pesticide mixtures using ms-PAF^13,31,32^. This study provides additional toxicity threshold data for alternative PSII and non-PSII herbicides for tropical marine species that can be incorporated in SSDs to improve WQGVs, including the merged DGVs applied to assess risk of pesticide mixtures to the GBR using ms-PAF methods^38^.

### Conclusion

Growth toxicity thresholds for PSII and non-PSII herbicides for microalgae *Tioschrysis lutea* and *Tetraselmis* sp. were determined, and both species of microalgae were over an order of magnitude more sensitive to PSII herbicides than non-PSII herbicides. When herbicides were tested individually, current PC99 PGV and DGV are adequate to protect both microalgal species, except from the PSII herbicide metribuzin which was harmful to *T. lutea* at concentrations lower than the PC99 PGV/DGV. However, since herbicides are rarely detected in isolation, it is important to consider other approaches, such as the ms-PAF, to assesses the cumulative ecological risk of pesticide mixtures. This study extends toxicity data for a suite of alternative herbicides detected in the GBRCA to further inform the development of national WQGVs and GBR-specific DGVs and associated ecological risk assessments for pesticide mixtures. This study targeted the more frequently detected alternate PSII and non-PSII herbicides, where more data was required to improve DGVs. However, there are more than 50 pesticides detected in the GBRCA without DGVs and further toxicity data is required for these pesticides to be included in future risk assessments for pesticide mixtures to the GBR.

## Methods

### Laboratory procedures

All test equipment (glass, HDPE) were acid washed (5% v/v nitric acid; Univar) for at least 24 h before being rinsed thrice with deionized, reverse-osmosis water and allowed to dry thoroughly. Erlenmeyer flasks (borosilicate, 125 mL) that served as test chambers for algal growth rate inhibition assays were additionally silanized with 2% dimethyldichlorosilane in 1,1,1-trichloroethane (Coatasil; Ajax Finechem) prior to acid-washing to minimize sorption of herbicides and algae to the glass.

### Microalgal culture conditions

*Tisochrysis lutea* (strain CS-177) and *Tetraselmis* sp. (strain CS-317) were obtained from the Australian National Algae Supply Service, Hobart (CSIRO). *Tetraselmis* sp. is a clonal strain described and tentatively named *Tetraselmis moretonica* sp. nov. in Mazid (2009)^73^, but further work is required to confirm this. Both species were cultivated as batch cultures using sterile 500 mL Erlenmeyer flasks that contained 300 mL of sterile EDTA-free Guillard’s f/2 marine medium^74^. Inoculum (5 mL) of 7-day-old cultures were aseptically transferred weekly to 300 mL of fresh sterile f/2 medium to maintain cultures in exponential growth. All culture flasks were swirled daily to resuspend and aerate algae. Cultures were maintained in plant growth chambers (Labec, model PG 36) at 28 ± 1 °C, 33 ± 1.5 psu, and 12:12 h light:dark cycle (80–100 μmol photons m^–2^ s^–1^, Sylvania Aquastar 39W).

### Preparation of test solutions

The toxicity of eleven and eight herbicides were tested with *T. lutea* and *Tetraselmis* sp., respectively. Herbicides tested for both species included six PSII herbicides simazine (CAS 122-34-9), tebuthiuron (CAS 34014-18-1), bromacil (CAS 314-40-9), metribuzin (CAS 21087-64-9), propazine (CAS 139-40-2) and diuron (CAS 330-54-1) as well as the acetyl-CoA carboxylase inhibitor haloxyfop-p-methyl (CAS 72619-32-0) and the acetohydroxyacid synthase inhibitor imazapic (CAS 104098-48-8). In addition, toxicity of the three auxin mimics, 2,4-D (CAS 94-75-7), MCPA (94-74-6) and fluroxypyr (CAS 69377-81-7) were assessed for *T. lutea*. Herbicide stock solutions (100–1000 mg L^−1^) were prepared in milli-Q water from analytical grade products (purity ≥ 98%, Sigma Aldrich, Castle Hill, NSW, Australia). Either dimethyl sulfoxide (DMSO) or acetone was used as a solvent carrier with the maximum amount in exposure not exceeding 0.02% (v/v) for DMSO and 0.04% (v/v) for acetone, except for the highest test concentrations for 2,4 D (≤ 0.4% (v/v) acetone), imazapic (≤ 0.1% (v/v) acetone) and simazine (0.25% (v/v) DMSO).

### Toxicity testing

Toxicity thresholds of the different herbicides for *T. lutea* and *Tetraselmis* sp. were determined by 72 h growth inhibition assays^48,49^. Test solutions for at least six different herbicide concentrations, including a seawater control (no herbicide), were prepared using filtered natural seawater (FSW, 0.5 µm), supplemented with quarter-strength EDTA-free f/2 media as a nutrient source^48^ (final concentration 1/8f). A separate experiment was performed to assess potential effects of the solvent carrier (DMSO or acetone) at the same concentration as used in the highest test treatment. Inoculum was taken from microalgae cultures in exponential growth phase (4–5 days old)^48,75^. Prior to inoculation, algae suspensions were centrifuged twice (*T. lutea*: 780×*g* for 7 min; *Tetraselmis* sp.: 240×*g* for 4 min), and algal pellets resuspended in FSW to remove any residual culture medium. Densities of the concentrated algal cultures were assessed via manual counts using a hemocytometer and test solutions were inoculated with a starting density of either 3 × 10^3^ or 1 × 10^4^ cells mL^−1^ for *T. lutea* and 2.5 × 10^3^ cells mL^−1^ for *Tetraselmis* sp., respectively. Following inoculation, each test solution was aliquoted into two or three replicate 125 mL Erlenmeyer flasks (50 mL test solution per flask) that were capped with a cotton plug and placed in a plant growth chamber (Labec, model PG36) set to 28 ± 1 °C and a 12:12 h light:dark cycle (80–100 μmol photons m^–2^ s^–1^, Sylvania Aquastar 39W). Test flasks were randomized and swirled daily to ensure sufficient gas exchange.

### Cell density measurements

Post 72 h exposure, subsamples (7 mL) were taken from each replicate flask and cell densities determined using a flow cytometer (BD Accuri C6, BD Biosciences, CA, USA) with a standard filter set as per Trenfield et al.^48^. The flow rate was set to 35 µL min^−1^, 16 µm core size and sample volume of 25 µL. Fixed gating was used around the viable (chlorophyll fluorescing) cells to avoid the counting of dead cells or non-microalgal particles. Samples were run in duplicates and the average count in the respective gated region was used to calculate the specific growth rate (SGR). SGR was expressed as the logarithmic increase in cell density over the exposure duration using the following Eq. (1):

1 $${{\text{SGR}}}_{\alpha -\beta }=\frac{{\text{ln}}\left({C}_{\beta }\right)- {\text{ln}}\left({C}_{\alpha }\right)}{{{\text{t}}}_{\upbeta }- {{\text{t}}}_{\mathrm{\alpha }}},$$

where SGR_α-β_ (day^−1^) is the specific growth rate between day α to β; C_α_ and C_β_ (cells mL^−1^) are the cell densities at time t_α_ and t_β_ (day), respectively^49^.

An assay was considered valid if the mean SGR of control samples was ≥ 0.92 day^−1^ and a coefficient of variation (CV) of mean SGR between controls was ≤ 10%^49^. At least two independent assays were performed for each species and herbicide combination.

### Water quality and chemical analyses

Physicochemical water quality parameters, including dissolved oxygen (mg L^−1^ and % saturation), pH, salinity (PSU) and electrical conductivity (mS cm^−1^), of all test solutions were measured at start (0 h) and at end of test (72 h) using a portable multi meter (HQ40D, Hach). Temperature was logged in 10-min intervals throughout the duration of tests (HOBO, Onset). At the end of the test, treatment replicate flasks were pooled after subsamples were taken for flow cytometry. Analytical samples (2 mL) were taken from pooled solutions and transferred into 4 mL amber glass vials before water quality measurements were conducted. Analytical samples were stored at − 20 °C before being transported to the Queensland Alliance for Environmental Health Sciences (QAEHS) at the University of Queensland for analysis. Prior to analysis, samples were spiked with surrogate labelled internal standards (Supplementary Table 3) for each herbicide at a final concentration of 10 ng mL^−1^. Limits of detection (LOD) and spike recoveries can be found in Supplementary Table 3. Blank samples (milli-Q water) were used for every 10–12 samples per batch. Herbicide analysis was performed using HPLC–MS/MS (SCIEX Triple Quad 6500 QTRAP mass spectrometer coupled to Shimadzu Nexera X2 uHPLC system) following the methodology provided in Thomas et al.^26^. Herbicide concentrations of 2–3 treatments per assay (including seawater control) were measured from the start and end of the test. To derive ‘measured’ concentrations for all other treatments, the geometric mean was calculated using the initial and final measured concentrations (time-weighted average). The average loss or difference from these measured concentrations was applied to all nominal concentrations.

### Statistical analyses

Specific growth rate for each treatment was expressed as percent inhibition relative to the control response. This allowed pooling of data from multiple assays that were performed for each herbicide and algae species combination. In order to generate concentration–response curves, measured herbicide concentrations were used and regression analyses were conducted following prescribed procedures^76^. The package DRC in R^77,78^ was used to model concentration–response relationships and estimate toxicity thresholds that inhibited 10% and 50% of the SGR relative to controls (EC10 and EC50, respectively). Regression models included log-logistic, Weibull and hormesis models of different levels of parametrization. Model comparisons were conducted using the Akaike Information Criterion (AIC) and the model that best described the data was applied to derive estimates of toxicity. The associated 95% confidence limits were estimated using the delta method.

A no effect concentration (NEC) value is the preferred measure of toxicity since NECs are more closely aligned with the objective of guideline values. The estimations of NEC values were performed using the Bayesian model fitting software JAGS^79^, via the R2jags^80^ and jagsNEC^81^ packages in R^82^. Proportional decline in SGR (1-inhibition) was modelled as a function of log measured concentration of each herbicide using a Bayesian non-linear gaussian model. This model has been specifically developed to derive NECs for a binomial response variable^83^ but can be more generally defined by the following Eq. (2):

2 $$E \left[Yi|xi\right]= \mu i = \alpha {\text{exp}}\left[-\beta \left(xi-\gamma \right)I\left(xi-\gamma \right)\right]-\Delta ,$$

where E[Y_i_|x_i_] is the mathematical expectation of Y_i_ (the response, e.g. in this case the proportional decline in SGR) conditional on a given concentration x_i_. The model parameters for the generalised case are *α* (the response at zero or low concentrations, also called “top”), − β (the rate of decay in the response after the NEC) and γ (the NEC value). For a gaussian *Y*, as used here, the model has the additional parameters Δ (an offset or intercept) and δ (the random error variance in *Y*). We used uninformative priors for the model parameters, including: *α* ~ dnorm(0, 0.1), β ~ dgamma(0.0001, 0.0001), y ~ dnorm(0, 0.01), Δ ~ dnorm(0, 0.1), and δ ~ dunif(0, 29). Note that in jags dnorm is parameterised as a mean and precision (rather than mean and sd, as in R). Models were run with 10,000 Markov chain Monte Carlo (MCMC) iterations after an initial “burn-in” period of 20,000 iterations and for five separate chains. Model fits were evaluated using trace plots and were found to have relatively good mixing in all cases. Bayesian 95% credible intervals (uncertainty) were based on the upper 97.5th and lower 2.5th percentile of the posterior sample.

### Supplementary Information

Supplementary Information.

## Data Availability

The datasets generated during the study are available in the eAtlas data repository at: https://eatlas.org.au/data/uuid/91967f34-b24d-4352-b6b0-526e54ec052f and https://eatlas.org.au/data/uuid/4a8d5927-0619-4f7e-8894-2e3aaf8d3aed.
