# Supplementary material for: Toxicity of herbicides to the marine microalgae Tisochrysis lutea and Tetraselmis sp
Source: Sci Rep. 2024 Jan 19;14:1727. doi: 10.1038/s41598-024-51401-3 (PMC10798944; doi:10.1038/s41598-024-51401-3)
Supplement: Supplementary file 1 — Supplementary Information. [file 41598_2024_51401_MOESM1_ESM.pdf]

Supplementary Information

Toxicity of herbicides to the marine microalgae *Tisochrysis lutea* and *Tetraselmis* sp.

Florita Flores[1,2*], Laura S. Stapp[3], Joost van Dam[3], Rebecca Fisher[4], Sarit Kaserzon[5] and Andrew P. Negri[1]

[1]Australian Institute of Marine Science, Townsville, Queensland 4810, Australia

[2]AIMS@JCU Division of Research and Innovation, Townsville, Queensland 4810, Australia

[3]Australian Institute of Marine Science, PO Box 41775, Casuarina, Northern Territory 0811, Australia

[4]Australian Institute of Marine Science, Indian Ocean Marine Research Centre, University of Western Australia, Crawley, Western Australia 6009, Australia

[5]Queensland Alliance for Environmental Health Sciences (QAEHS), The University of Queensland, Woolloongabba, Queensland 4102, Australia

Correspondence:

PMB No. 3, Townsville MC, Townsville QLD 4810, Australia

*f.flores@aims.gov.au

Supplementary Table 1. Measured physico-chemical parameters (pH, salinity, conductivity (Cond), dissolved oxygen (DO), temperature (Temp)) and measured concentrations from each *Tisochrysis lutea* growth inhibition assay. TL = *Tisochrysis lutea*, FL = fluroxypyr, HA = haloxyfop, DI = diuron, ME = metribuzin, IM = imazapic, TB = tebuthiuron, SI = simazine, BR = bromacil, PR = propazine, 24D = 2,4-D, MC = MCPA.

				Test initiation (day 0)					Test finalisation (day 3)					
Herbicide	Test ID	Nominal concentration (µg L^{-1})	Measured concentration (µg L^{-1})	pH	Salinity (PSU)	Cond (mS cm^{-1})	DO (mg L^{-1})	DO (% sat)	pH	Salinity (psu)	Cond (mS cm^{-1})	DO (mg L^{-1})	DO (% sat)	Temp (°C)
Fluroxypyr	TLFL01	0	0	8.1	31.8	47.5	8.1	96	8.9	33.1	52.1	12	150	N/A
		1	0.63	8.3	31.8	47.8	7.9	94	8.9	33	51.4	11.3	140	
		3	1.89	8.3	31.8	47.8	7.9	94	8.9	33	51.3	11.1	137	
		10	6.30	8.3	31.8	47.9	7.9	94	9	32.8	50.9	11.7	144	
		30	18.9	8.3	31.8	47.8	7.9	95	8.9	33	51.1	11.6	143	
		100	63.0	8.3	32.4	48.5	8	95	8.9	33.5	52	11.3	140	
	TLFL02	0	0	8.2	32.8	49.4	7.8	93	8.8	34.1	52	12.7	155	28.5 ± 1.0
		30	18.9	8.2	32.7	49.1	7.9	94	8.8	34.3	52	11.7	141	
		100	63.0	8.2	33	49.2	7.9	94	8.7	34.3	52	11.9	144	
		300	189	8.2	33	49.2	7.9	94	8.7	34.3	52	12	145	
		1000	630	8.2	33.1	49.3	7.9	94	8.7	34.3	52	12	145	
		3000	1890	8.3	33.1	49	7.9	94	8.7	34.3	51.6	12.4	149	
	TLFL03	0	0	8.0	32.7	48.4	8.4	99	8.6	34	52.6	12.1	148	27.9 ± 0.3
		500	315	8.0	32.7	48.5	8.5	100	8.5	34	52.8	11.9	146	
		1000	630	8.0	32.7	48.6	8.4	100	8.5	33.9	52.5	11.3	138	
		2000	1260	8.0	32.7	48.5	8.5	100	8.5	33.9	52.3	11.5	140	
		5000	3150	8.0	32.7	48.5	8.4	100	8.5	33.9	52.2	11.2	137	
		10000	6300	8.0	32.7	48.5	8.5	101	8.5	33.9	52.1	11.1	136	
Haloxyfop	TLHA01	0	0	8.1	30.8	46.1	8.5	101	8.6	32.2	49.3	13.3	163	28.1 ± 0.7
		10	9.57	8.1	30.8	46.4	8.5	101	8.7	32.1	48.8	13.1	161	
		30	28.7	8.1	30.8	46.5	8.4	101	8.7	32.1	49.8	13.1	160	
		100	95.7	8.1	30.8	46.6	8.4	100	8.7	31.8	48.9	13	158	

		300	287	8.2	30.8	46.5	8.4	100	8.7	32.2	50	13.1	161	
		1000	957	8.2	30.7	46.5	8.4	100	8.7	32	49.8	12.3	151	
		2000	1914	8.2	30.7	46.5	8.4	100	8.7	31.9	49.1	12.1	147	
		3000	2872	8.2	30.7	46.5	8.4	101	8.7	32	49.2	11.9	144	
	TLHA02	0	0	8.2	31.7	47.7	8.4	100	8.6	32.9	51.1	12.3	152	
		500	479	8.2	31.7	47.9	8.4	101	8.7	33	51.5	13.4	165	
		2000	1914	8.2	31.7	47.8	8.4	100	8.7	32.9	51.4	13.3	164	
		3000	2872	8.2	32.2	48.5	8.4	101	8.7	33.4	52	12	147	28.0 ± 1.4
		4000	3829	8.2	31.7	47.9	8.3	100	8.6	32.9	50.6	12	146	
		5000	4786	8.2	31.7	47.8	8.3	100	8.3	32.9	51	8.4	103	
		7500	7179	8.2	31.7	47.9	8.4	100	8.2	32.8	50.9	8.3	102	
	TLDI01	0	0	8.0	31.4	46.9	7.8	93	7.9	32	48.9	8.1	98	
		1	0.84	7.9	31.4	46.8	7.9	94	8	31.7	48.7	8	97	
		3	2.51	8.0	31.4	46.7	7.9	94	8	31.6	48.8	7.9	96	28.6 ± 0.2
		10	8.37	8.0	31.4	46.6	7.9	94	8	31.5	48.2	7.8	95	
		30	25.1	8.0	31.4	46.8	7.9	94	8	31.5	48	7.9	95	
		100	83.7	8.0	31.3	46.5	7.9	94	8	31.5	47.7	7.9	95	
	TLDI02	0	0	8.1	29.6	45.5	7.8	95	8.1	31	48.2	8	98	
		0.3	0.3	7.9	29.6	45.2	7.8	94	8.2	31	48.2	7.9	97	
		0.6	0.5	8.0	29.6	45.2	7.8	94	8.2	31.2	48.9	8	98	28.5 ± 0.3
Diuron		2	1.7	7.9	29.6	45.2	7.9	95	8.2	31.2	48.9	7.8	96	
		6	5.0	8.0	29.6	45.1	7.9	95	8.3	31	47.7	7.9	96	
		12	10.0	7.9	29.6	44.9	7.9	95	8.2	31.2	48	7.8	95	
	TLDI03	0	0	8.3	29.2	43.6	7.9	94	8.3	31	46.1	8.3	98	
		0	0	8.2	29.3	43.8	8	94	8.3	31.1	49.2	8.2	99	
		0.5	0.42	8.3	29.1	43.7	8	95	8.4	30.9	48.7	7.8	96	28.5 ± 0.4
		0.8	0.67	8.2	29.2	43.9	8	95	8.3	30.7	47.6	7.8	95	
		1.5	1.26	8.3	29.2	43.8	8	95	8.4	30.5	47.1	7.9	96	
		2.5	2.09	8.2	29.2	43.7	8	95	8.2	30.6	47.2	7.9	96	
	TLME01	0	0	8.0	31.4	46.8	7.8	93	7.9	32	48.9	8.1	98	
Metribuzin		1	1.08	8.0	31.4	46.5	7.9	94	8	31.8	48.8	8	97	28.6 ± 0.2
		3	3.23	8.0	31.4	46.5	7.9	94	8.1	31.8	48.7	7.8	95	

		10	10.8	8.0	31.4	46.6	7.9	94	8	31.7	48.1	7.9	95	
		30	32.3	8.0	31.3	46.5	7.9	94	8.1	31.6	47.8	7.9	95	
		100	108	8.0	31.4	46.5	7.9	94	8	31.6	47.6	7.9	95	
	TLME02	0	0	8.1	29.6	45.5	7.8	95	8.1	31	48.2	8	98	28.5 ± 0.3
		0.3	0.3	8.0	29.7	45.2	7.9	95	8.3	31	47.2	8	96	
		0.6	0.6	7.9	29.7	45.4	7.9	95	8.3	31.1	47.6	8.1	98	
		2	2.2	8.0	29.6	45.4	7.9	95	8.3	31.1	47.8	7.9	96	
		6	6.5	7.9	29.6	45.4	7.8	95	8.3	31.1	48	7.9	96	
		12	12.9	8.0	29.5	45	7.9	95	8.3	31	47.6	7.8	95	
	TLME03	0	0	8.3	29.2	43.6	7.9	94	8.3	31	46.1	8.3	98	28.5 ± 0.4
		0.2	0.22	8.3	29.3	44.1	7.9	94	8.3	31.1	49.1	7.9	97	
		0.5	0.54	8.2	29.4	44.4	7.9	95	8.3	30.7	48.6	7.8	96	
		0.8	0.86	8.3	29.2	44.3	7.9	95	8.3	30.5	47.6	7.8	96	
		1.5	1.61	8.3	29.2	44.2	7.9	94	8.3	30.6	47.4	7.8	95	
		5	5.38	8.3	29.2	44	8	95	8.3	30.5	47.2	7.8	95	
Imazapic	TLIM01	0	0	8.0	31.4	46.9	7.8	93	7.9	32	48.9	8.1	98	28.6 ± 0.2
		1	1.58	8.0	31.4	46.6	7.9	94	8.1	31.8	48.7	8.1	98	
		3	4.73	8.0	31.4	46.8	7.9	94	8	31.7	48.7	8	98	
		10	15.8	8.0	31.4	46.5	7.9	94	8.1	31.7	48	8	97	
		30	47.3	8.0	31.4	46.5	8	94	8.1	31.9	48.8	8	98	
		100	158	8.0	31.4	46.4	7.9	94	8.1	31.6	48.3	8	97	
	TLIM02	0	0	8.1	29.6	45.5	7.8	95	8.1	31	48.2	8	98	28.5 ± 0.3
		30	47.3	8.0	29.6	45	7.9	95	8.3	30.9	47.9	8	97	
		100	158	8.0	29.1	44.4	7.9	95	8.3	30.5	47.6	8.1	99	
		300	473	7.9	29.6	45.4	7.9	95	8.3	31	47.6	8	97	
		1000	1575	8.0	29.6	48.2	7.8	95	8.4	30.9	47.3	7.9	96	
		3000	4725	8.0	29.5	44.9	7.8	95	8.3	31.1	47.7	7.9	95	
	TLIM03	0	0	8.3	29.2	43.6	7.9	94	8.3	31	46.1	8.3	98	28.5 ± 0.4
		100	158	8.2	29.2	43.8	8	95	8.3	30.5	46.8	7.9	96	
		500	788	8.3	29.2	43.9	8	95	8.3	30.4	46.7	8	97	
		2000	3150	8.2	29.2	43.9	8	95	8.3	30.8	47.7	7.9	96	
		5000	7875	8.3	29.1	43.7	8	95	8.3	30.5	47.7	7.8	96	

		10000	15750	8.2	27.6	41.5	8	95	8.3	29	44.9	7.9	95	
Tebuthiuron	TLTB01	0	0	8.2	33	49	8.6	100	8.6	34.8	54.1	11.4	140	28.6 ± 0.2
		1	1.04	8.2	33	49.1	8.6	101	8.6	34.4	53.5	11.5	141	
		3	3.13	8.2	33	49.1	8.6	101	8.6	34.6	54.3	11.1	137	
		10	10.4	8.2	33	49.1	8.6	101	8.6	34.5	53.8	10.6	131	
		30	31.3	8.2	33	49.2	8.5	101	8.5	34.4	53.6	10.1	125	
		100	104	8.2	33	49.2	8.5	101	8.3	34.4	53.7	8.53	105	
		300	313	8.2	33	49.2	8.5	101	8.3	34.3	53.1	8.2	100	
		1000	1043	8.2	33	49.2	8.5	101	8.2	34.4	52.9	8.1	100	
	TLTB02	0	0	8.2	31.4	47.1	8.7	101	8.6	32.9	51.6	11.8	146	29.7 ± 0.3
		5	5.2	8.2	31.4	47.1	8.4	100	8.5	32.8	51	11.6	142	
		15	15.6	8.2	31.4	47.1	8.4	100	8.5	32.6	50.5	11.4	138	
		25	26.1	8.2	31.4	47.1	8.4	100	8.5	32.8	51.2	11.3	138	
		50	52	8.2	31.5	47.1	8.4	100	8.4	32.8	51.8	9.7	120	
		80	83	8.2	31.5	47.1	8.5	100	8.3	32.8	51.4	8.9	110	
		130	136	8.2	31.4	47	8.4	100	8.3	32.7	51	8.4	103	
		200	209	8.2	31.3	46.8	8.5	100	8.2	32.6	50.3	8.2	100	
Simazine	TLSI01	0	0	8.2	31.7	47.5	8.3	99	8.6	33.7	51.8	12.1	148	27.6 ± 0.6
		1	1.18	8.2	31.6	47.4	8.3	99	8.6	33.5	53	11.7	147	
		3	3.53	8.2	31.5	47.2	8.4	99	8.6	32.9	52	11.1	139	
		10	11.8	8.2	31.7	47.4	8.4	99	8.6	33.1	52.2	11.7	146	
		30	35.3	8.2	31.6	47.3	8.4	99	8.5	32.9	51.9	10.4	129	
		100	118	8.2	31.8	47.6	8.3	99	8.4	33.1	51.9	9.4	116	
		300	353	8.2	31.6	47.4	8.3	99	8.3	32.8	51.2	8.3	102	
		1000	1176	8.2	31.7	47.5	8.3	99	8.3	32.9	51.1	8.3	102	
	TLSI02	0	0	8.2	33	49.4	8.4	100	8.5	34.9	53.3	11.1	135	29.7 ± 0.3
		5	5.9	8.2	32.8	49	8.5	100	8.5	34.4	52.9	10.7	129	
		15	17.6	8.2	32.8	49.1	8.4	101	8.5	34.3	53.6	10.5	129	
		25	29.4	8.2	32.8	49	8.5	101	8.5	34.2	53.2	10.3	126	
		50	58.8	8.2	32.8	49.1	8.4	100	8.4	34.1	52.9	9.8	120	
		80	94	8.2	32.8	49.1	8.4	100	8.4	34.2	52.6	9	109	
		130	153	8.2	32.8	49	8.4	100	8.3	34.1	52.5	8.7	106	

		600	706	8.2	32.8	49.1	8.4	100	8.3	34.1	52.1	8.3	100	
Bromacil	TLBR01	0	0	8.2	32	47.1	8.1	96	8.7	33.8	51.5	10.2	123	28.4 ± 0.2
		1	0.62	8.3	32.1	48	8	95	9	33.9	53.5	10.8	135	
		3	1.87	8.3	32.1	48.2	8	95	8.9	33.8	53.5	10.6	133	
		10	6.22	8.3	32.1	48.3	8	96	8.4	33.6	52.8	8.5	106	
		30	18.7	8.3	32.1	48.2	8	95	8.4	33.8	53.6	7.7	96	
		100	62.2	8.3	32.1	47.9	8	95	8.3	33.8	54.2	7.6	97	
	TLBR02	0	0	8.1	30.4	45	8.7	102	8.6	32.1	50.3	9.1	114	28.4 ± 0.3
		2	1.24	8.2	30.4	45.2	8.4	99	8.5	31.7	49.8	8.5	105	
		4	2.49	8.2	30.4	45.3	8.4	99	8.4	31.8	49.8	8.1	100	
		6	3.73	8.2	30.5	45.3	8.4	99	8.4	31.8	50.1	7.9	98	
		15	9.33	8.2	30.4	45.3	8.3	98	8.4	31.7	49.7	7.7	96	
		20	12.45	8.2	30.4	45.4	8.4	99	8.3	31.7	49.5	7.7	95	
	TLBR03	0	0	8.1	31.5	46.9	7.9	93	8.6	32.6	50	8.6	105	28.6 ± 1.2
		0.5	0.31	8.3	31.5	47.5	7.8	94	8.6	32.6	49.9	8.6	104	
		1.5	0.93	8.3	31.6	48.1	7.8	94	8.6	32.7	50	8.6	105	
		5	3.11	8.3	31.6	48.4	7.7	94	8.5	32.7	50.1	8.1	99	
		12	7.47	8.3	31.6	48.3	7.8	94	8.5	32.7	50.1	8.1	99	
		50	31.12	8.3	31.5	47.8	7.7	93	8.4	32.5	49.5	7.9	95	
Propazine	TLPR01	0	0	8.2	32	47.1	8.1	96	8.7	33.8	51.5	10.2	123	28.4 ± 0.2
		1	0.92	8.3	32.1	47.7	8	95	8.8	33.9	54.5	9.5	120	
		3	2.76	8.3	32.1	47.9	8	95	8.9	33.8	53.7	10	126	
		10	9.21	8.3	32.1	47.9	8	95	8.8	34	54.4	10	127	
		30	27.6	8.3	32.1	47.9	8	95	8.6	33.8	53.6	8.9	112	
		100	92.1	8.3	32.1	47.7	7.9	94	8.4	33.8	53.2	7.8	98	
	TLPR02	0	0	8.1	30.4	45	8.7	102	8.6	32.1	50.3	9.1	114	28.4 ± 0.3
		20	18.4	8.2	30.4	45.4	8.3	98	8.4	31.9	49.7	8	98	
		40	36.8	8.2	30.4	45.2	8.4	99	8.4	32	50.1	7.8	97	
		60	55.2	8.2	30.4	45.4	8.3	99	8.3	31.9	49.6	7.7	96	
		80	73.7	8.2	30.4	45.3	8.3	99	8.3	31.9	49.5	7.7	95	
		150	138	8.2	30.4	45.3	8.3	98	8.3	31.9	49.2	7.7	95	
	TLPR03	0	0	8.1	31.5	46.9	7.9	93	8.6	32.6	50	8.6	105	

		5	4.60	8.3	30.6	46	7.8	93	8.6	32	48.8	8.5	102	28.6 ± 1.2
		15	13.8	8.3	31.5	47.5	7.8	94	8.6	32.9	50.1	8.4	101	
		25	23.0	8.3	31.6	47.9	7.8	94	8.5	32.8	49.9	8.2	99	
		125	115	8.3	31.6	48	7.8	94	8.4	32.8	49.7	7.9	95	
		300	276	8.3	31.5	47.7	7.8	94	8.3	32.9	49.8	7.8	94	
2,4-D	TL24D01	0	0	8.2	32	47.1	8.1	96	8.7	33.8	51.5	10.2	123	28.4 ± 0.2
		1	1.17	8.3	32.1	47.8	8.1	96	8.9	33.8	52.2	9.9	122	
		3	3.51	8.3	32.1	48.1	8.1	96	8.8	33.7	52.4	10.1	124	
		10	11.7	8.3	32.1	48	8	96	8.8	33.8	53.4	9.6	117	
		30	35.1	8.3	32.1	48	8	95	8.8	33.5	52.3	9.9	122	
		100	117	8.3	32.1	48	8	95	8.8	33.5	51.3	9.9	121	
	TL24D02	0	0	8.1	30.4	45	8.7	102	8.6	32.1	50.3	9.1	114	28.4 ± 0.3
		30	35.1	8.2	30.5	45.2	8.5	101	8.6	31.8	50.2	8.8	110	
		100	117	8.2	30.4	45.2	8.5	100	8.6	31.8	50.3	8.6	107	
		300	351	8.2	30.5	45.3	8.4	99	8.6	31.6	49.8	8.4	104	
		1000	1170	8.2	30.4	45.3	8.3	98	8.6	31.7	50.2	8.2	102	
		3000	3510	8.2	30.4	45.3	8.3	98	8.5	31.8	50	8.1	101	
	TL24D03	0	0	8.1	31.5	46.9	7.9	93	8.6	32.6	50	8.6	105	28.7 ± 0.9
		500	585	8.3	31.6	47.3	7.9	95	8.6	32.7	50.4	8.7	106	
		1000	1170	8.3	31.6	47.6	7.9	94	8.6	32.8	50.4	8.7	106	
		2000	2340	8.3	31.5	47.7	7.8	94	8.6	32.6	50.1	8.8	107	
		5000	5850	8.3	31.5	47.7	7.8	94	8.6	32.7	50.1	8.8	107	
		10000	11700	8.2	31.4	47.3	7.9	95	8.6	32.9	50.2	8.6	104	
	TL24D04	0	0	8.1	31.8	47.5	8.1	96	8.9	33.1	52.1	12	150	No data
		31600	36972	8.2	31.4	47.1	7.9	94	8.6	32.9	50.6	8.4	102	
		56000	65521	8.0	31.6	47.3	7.9	95	8.5	32.9	50.4	8	98	
MCPA	TLMC01	0	0	8.1	31.8	47.5	8.1	95	8.9	33.1	52.1	12	150	No data
		1	1.98	8.3	31.8	47.7	7.9	94	9	33	51.1	11	136	
		3	5.93	8.3	31.8	47.5	7.9	95	8.9	33	51.2	11	135	
		10	19.8	8.3	31.9	47.8	7.9	95	9	33.2	51.4	11.5	142	
		30	59.3	8.3	31.8	47.8	7.9	95	8.9	33.1	51.3	10.8	133	
		100	198	8.3	31.8	47.7	7.9	95	8.9	33	50.9	11.2	137	

	TLMC02	0	0	8.0	32.7	48.4	8.4	99	8.6	34	52.6	12.1	148	
		30	59.3	8.0	32.8	48.6	8.3	99	8.5	34.1	51.5	12.1	145	
		100	198	8.1	32.7	48.6	8.3	98	8.5	33.9	51.5	11.7	140	
		300	593	8.0	32.7	48.7	8.3	99	8.5	33.9	51.5	11.6	140	
		500	989	8.0	32.7	48.7	8.3	99	8.5	33.4	50.5	11.4	137	27.9 ± 0.3
		1000	1978	8.0	32.8	48.6	8.4	99	8.5	33.8	51.3	11.1	134	
		2000	3955	8.0	32.8	48.7	8.4	100	8.5	34.1	50.8	11.2	133	
		3000	5933	8.0	32.7	48.6	8.4	99	8.5	33.8	50.8	11	131	
		5000	9888	8.0	32.7	48.6	8.4	100	8.5	33.9	50.8	10.8	129	
		10000	19775	8.0	32.4	48.1	8.4	100	8.4	33.2	49.7	10.9	129	

Supplementary Table 2. Measured physico-chemical parameters (pH, salinity, conductivity (Cond), dissolved oxygen (DO), temperature (Temp)) and measured concentrations from each *Tetraselmis* sp. growth inhibition assay. TS = *Tetraselmis* sp., HA = haloxyfop, DI = diuron, ME = metribuzin, IM = imazapic, TB = tebuthiuron, SI = simazine, BR = bromacil, PR = propazine.

				Test initiation (day 0)					Test finalisation (day 3)					
Herbicide	Test ID	Nominal concentration (µg L^{-1})	Measured concentration (µg L^{-1})	pH	Salinity (PSU	Cond (mS cm^{-1})	DO (mg L^{-1})	DO (% sat)	pH	Salinity (psu)	Cond (mS cm^{-1})	DO (mg L^{-1})	DO (% sat)	Temp (°C)
Haloxyfop	TSHA01	0	0	8.2	32.6	49.1	8.4	100	8.7	33.7	51.2	12.8	154	
		1	0.91	8.2	32.7	48.9	8.4	100	8.6	33.7	50.5	11.7	140	
		3	2.74	8.2	32.7	49	8.4	100	8.7	33.7	50.1	12.3	157	
		10	9.13	8.2	32.7	49	8.4	100	8.7	33.7	50.2	11.8	141	26.7 ± 1.5
		30	27.4	8.2	32.7	49	8.4	100	8.6	33.7	50	12	142	
		100	91.3	8.2	32.7	48.9	8.4	100	8.6	33.5	49.3	11.9	140	
		300	274	8.2	32.7	48.9	8.4	101	8.7	33.9	50.9	12.5	149	
		1,000	913	8.2	32.3	49.8	8.4	100	8.6	34.2	50.5	11.7	138	
	TSHA02	0	0	8.1	31.8	47.5	8.6	102	8.6	33.3	50.8	12.1	147	
		500	457	8.1	31.7	47.7	8.4	100	8.7	33.1	52.2	12	149	27.9 ± 1.4
		2000	1826	8.2	31.5	47.5	8.4	101	8.7	32.5	50.9	11.9	147	

		3000	2739	8.2	31.6	47.7	8.4	101	8.6	32.8	50.8	11.5	142	
		4000	3652	8.2	31.6	47.7	8.4	100	8.6	32.9	51.1	10.5	130	
		5000	4565	8.2	31.7	47.7	8.3	100	8.4	32.6	49.7	9.6	116	
		7500	6848	8.2	31.6	47.7	8.3	100	8.3	32.7	49.7	8.8	106	
		10000	9131	8.2	31.6	47.6	8.4	100	8.3	32.9	50.2	8.5	108	
Diuron	TSDI01	0	0	8.2	32.6	49.1	8.4	100	8.7	33.7	51.2	12.8	154	27.3 ± 0.6
		1	0.92	8.2	32.7	48.9	8.4	100	8.6	33.7	50.5	11.7	140	
		3	2.75	8.2	32.7	49	8.4	100	8.7	33.7	50.1	12.3	157	
		10	9.16	8.2	32.7	49	8.4	100	8.7	33.7	50.2	11.8	141	
		30	27.5	8.2	32.7	49	8.4	100	8.6	33.7	50	12	142	
		100	91.6	8.2	32.7	48.9	8.4	100	8.6	33.5	49.3	11.9	140	
		300	275	8.2	32.7	48.9	8.4	101	8.7	33.9	50.9	12.5	149	
		1000	916	8.2	32.3	49.8	8.4	100	8.6	34.2	50.5	11.7	138	
	TSDI02	0	0	8.1	31.8	47.5	8.6	102	8.6	33.3	50.8	12.1	147	27.5 ± 0.5
		0.3	0.27	8.1	31.7	47.7	8.4	100	8.7	33.1	52.2	12	149	
		0.6	0.55	8.2	31.5	47.5	8.4	101	8.7	32.5	50.9	11.9	147	
		2	1.83	8.2	31.6	47.7	8.4	101	8.6	32.8	50.8	11.5	142	
		4	3.66	8.2	31.6	47.7	8.4	100	8.6	32.9	51.1	10.5	130	
		6	5.50	8.2	31.7	47.7	8.3	100	8.4	32.6	49.7	9.6	116	
		12	11.0	8.2	31.6	47.7	8.3	100	8.3	32.7	49.7	8.8	106	
		20	18.3	8.2	31.6	47.6	8.4	100	8.3	32.9	50.2	8.5	108	
Metribuzin	TSME01	0	0	8.1	32.4	48.2	8.7	102	8.5	33.5	51.1	10.6	128	27.3 ± 0.6
		1	1.39	8.2	32.4	48.2	8.5	100	8.5	33.5	51.4	10.9	132	
		3	4.18	8.2	32.4	48.3	8.5	100	8.5	33.5	51.3	10.3	124	
		10	13.9	8.2	32.4	48.4	8.5	101	8.3	33.5	51.2	8.8	107	
		30	41.8	8.2	32.3	48.4	8.4	100	8.3	33.5	51.1	8.5	102	
		100	139	8.2	32.3	48.3	8.4	100	8.3	33.5	51.1	8.5	102	
		300	418	8.2	32.3	48.2	8.4	100	8.3	33.3	50.9	8.4	102	
		1000	1394	8.2	32.4	48.3	8.5	101	8.3	33.9	50.3	8.4	102	
	TSME02	0	0	8.2	32.4	49.3	8.3	100	8.6	33.7	52.6	11.9	147	28.0 ± 0.7
		0.5	0.70	8.2	32.4	49.1	8.4	101	8.6	33.6	52.6	11.7	146	
		2	2.79	8.2	32.5	49.3	8.4	101	8.6	33.6	52.4	11.5	141	

		4	5.58	8.2	32.5	49.3	8.4	101	8.5	33.7	53.1	10.1	125	
		6	8.36	8.2	32.5	49.3	8.3	100	8.4	33.7	53.1	9.4	117	
		8	11.2	8.2	32.4	49.3	8.3	100	8.3	33.6	53.2	9	113	
		12	16.7	8.2	32.5	49.4	8.3	100	8.3	33.5	52.7	8.6	106	
		15	20.9	8.2	32.4	49.4	8.3	101	8.2	33.6	52.5	8.4	105	
Imazapic	TSIM01	0	0	8.2	32	48.7	8.3	100	8.6	33.6	52.9	11.3	140	28.0 ± 0.3
		3	6.23	8.2	32.6	49.5	8.4	100	8.6	34.2	53.5	11.6	144	
		10	20.8	8.2	32	48.7	8.4	101	8.6	33.4	52.2	11.8	145	
		30	62.3	8.2	32	48.7	8.3	100	8.6	33.6	52.8	11.6	143	
		100	208	8.2	32	48.7	8.3	100	8.6	33	51.3	11.3	139	
		300	623	8.2	32	48.7	8.3	101	8.6	33.2	51.6	11.3	139	
		1000	2075	8.2	32	48.9	8.3	100	8.6	33	50.8	10.9	133	
		3000	6225	8.2	32	48.7	8.3	100	8.6	33.4	51.6	11.1	136	
	TSIM02	0	0	8.1	32.5	49	8.5	102	8.5	34.1	52.5	11.3	139	27.5 ± 0.5
		500	1038	8.2	32.4	49.2	8.4	101	8.5	33.7	52	11.1	136	
		2000	4150	8.2	32.5	49.2	8.4	102	8.5	33.5	51.2	11.1	135	
		3000	6225	8.2	32.5	49.4	8.4	102	8.6	33.5	51.2	11.1	134	
		4000	8300	8.1	32.5	49.3	8.4	101	8.5	33.6	51.5	10.8	132	
		5000	10375	8.2	32.4	49.3	8.4	101	8.6	33.6	51.7	10.9	133	
		7500	15563	8.2	32.4	49.3	8.4	101	8.5	33.4	50.8	10.8	130	
		10000	20750	8.2	32.4	49.3	8.4	101	8.5	33	50	10.9	131	
Tebuthiuron	TSTB03	0	0	8.2	32	48.7	8.3	100	8.6	33.6	52.9	11.3	140	28.0 ± 0.3
		5	4.38	8.2	32	48.8	8.3	100	8.6	33.5	52.9	11.4	142	
		15	13.1	8.2	32	48.8	8.3	100	8.5	33.4	53.1	10.6	133	
		25	21.9	8.2	32	48.8	8.3	100	8.5	33.5	53.2	10.2	128	
		50	43.8	8.2	32	48.7	8.3	100	8.4	33.3	52.2	9.1	113	
		80	70.1	8.2	32	48.8	8.3	100	8.3	33.1	51.4	8.5	105	
		130	114	8.2	32	48.8	8.3	100	8.3	33.3	51.6	8.4	102	
		600	526	8.2	32	48.8	8.3	100	8.3	33.3	51.8	8.2	101	
	TSTB04	0	0	8.2	32.4	49.3	8.3	100	8.6	33.7	52.6	11.9	147	28.0 ± 0.7
		3	2.6	8.2	32.5	49.3	8.4	101	8.6	33.6	53.1	11	137	
		10	8.8	8.2	32.5	49.4	8.4	101	8.6	33.8	53.7	10.8	135	

		20	17.5	8.2	32.5	49.2	8.3	101	8.5	33.7	53.7	10.3	130	
		30	26.3	8.2	32.5	49.4	8.4	101	8.4	33.5	53.1	9.7	121	
		40	35.0	8.2	32.5	49.5	8.3	100	8.3	33.6	52.4	9.1	112	
		100	87.6	8.2	32.5	49.4	8.3	101	8.3	33.7	53.4	8.3	104	
		300	263	8.2	32.4	49.4	8.3	101	8.2	33.6	52.8	8.2	101	
Simazine	TSSI02	0	0	8.2	32.6	49.7	8.3	101	8.5	34.1	52.6	10.5	129	28.0 ± 0.3
		5	6.26	8.2	32.7	49.9	8.3	101	8.5	34.3	53.3	10.1	124	
		15	18.8	8.2	32.7	49.9	8.3	101	8.4	34	52.7	9.9	122	
		25	31.3	8.2	32.7	49.9	8.3	100	8.4	34.1	52.8	9.6	117	
		50	62.6	8.2	32.7	49.8	8.3	100	8.4	33.9	52.6	9	111	
		80	100	8.2	32.7	49.9	8.3	100	8.3	34.2	53.3	8.7	107	
		130	163	8.2	32.7	49.9	8.3	100	8.3	34	53	8.3	103	
		600	751	8.2	32.7	49.8	8.3	100	8.3	33.8	52.3	8.2	100	
	TSSI04	0	0	8.2	32.6	49	8.5	101	8.6	33.9	51.3	12	145	26.4 ± 1.1
		3	3.8	8.2	32.6	48.9	8.4	100	8.6	33.8	50.5	11.4	136	
		10	12.5	8.2	32.6	49.9	8.5	101	8.6	33.9	51	11.8	142	
		20	25.0	8.2	32.6	48.8	8.4	100	8.6	33.8	50.7	11.4	134	
		30	37.6	8.2	32.7	48.9	8.4	100	8.5	33.9	51	11	132	
		40	50	8.2	32.7	49	8.4	100	8.5	33.9	51	10.4	125	
		100	125	8.2	32.7	49	8.4	100	8.3	33.9	51	8.9	108	
		300	376	8.2	32.7	49	8.4	100	8.2	33.9	50.9	8.6	103	
Bromacil	TSBR01	0	0	8.1	32.4	48.1	8.7	102	8.5	33.5	51.1	10.6	128	27.3 ± 0.6
		1	0.67	8.1	32.4	48.2	8.5	101	8.4	33.7	51.7	9.9	120	
		3	2.0	8.2	32.4	48.3	8.5	100	8.4	33.7	51.9	9.5	116	
		10	6.67	8.2	32.4	48.2	8.4	100	8.3	33.7	51.8	8.7	106	
		30	20.0	8.2	32.4	48.4	8.4	100	8.3	33.6	51.6	8.4	102	
		100	66.7	8.2	32.4	48.3	8.5	100	8.3	33.7	51.9	8.3	101	
		300	200	8.2	32.4	48.4	8.5	100	8.3	33.6	51.4	8.4	101	
		1000	667	8.2	32.3	48.2	8.4	100	8.3	33.7	51.6	8.3	101	
	TSBR02	0	0	8.1	32.5	49	8.5	102	8.5	34.1	52.5	11.3	139	27.5 ± 0.5
		0.3	0.20	8.1	32.5	49.2	8.5	102	8.5	33.7	52.1	10.5	129	
		0.6	0.40	8.1	32.5	49.2	8.4	101	8.5	33.8	52.6	10.3	127	

		2	1.33	8.2	32.5	49.1	8.4	101	8.4	33.5	51.6	9.8	119	
		4	2.67	8.1	32.5	49.1	8.4	100	8.4	33.7	52	9.4	115	
		6	4.00	8.2	32.5	49.2	8.4	101	8.3	33.7	51.7	8.9	109	
		12	8.00	8.2	32.5	49.3	8.4	101	8.3	33.6	51.8	8.5	104	
		20	13.3	8.2	32.5	49.3	8.4	101	8.2	33.7	51.8	8.4	102	
Propazine	TSPR01	0	0	8.2	32.6	49.1	8.4	100	8.7	33.7	51.2	12.8	154	26.7 ± 1.5
		1	0.96	8.2	32.6	49	8.4	100	8.7	33.6	50	11.9	143	
		3	2.88	8.2	32.7	49.1	8.4	100	8.7	33.7	50.2	12.2	146	
		10	9.61	8.2	32.7	49.1	8.4	100	8.6	33.6	50	12.3	147	
		30	28.8	8.2	32.7	48.9	8.4	100	8.6	34	51.6	11.6	147	
		100	96.1	8.2	32.7	49.6	8.4	100	8.3	33.6	50.5	9.1	110	
		300	288	8.2	32.7	49	8.4	100	8.2	33.7	50.6	8.6	104	
		1000	961	8.2	32.7	49.1	8.4	100	8.2	33.7	50.9	8.6	103	
	TSPR02	0	0	8.1	31.8	47.5	8.6	102	8.6	33.3	50.8	12.1	147	27.9 ± 1.4
		5	4.8	8.2	31.7	47.7	8.4	100	8.7	33.2	51.8	12.5	154	
		15	14.4	8.2	31.7	47.7	8.4	100	8.6	32.8	50.8	11.6	143	
		25	24.0	8.2	31.7	47.6	8.4	100	8.6	33.2	52.4	11.3	141	
		40	38.4	8.2	31.7	47.7	8.4	100	8.5	32.8	51.6	10.5	131	
		50	48.1	8.2	31.7	47.8	8.4	101	8.5	33	51.9	10	124	
		200	192	8.2	31.7	47.7	8.4	101	8.2	32.8	50.6	8.4	104	
		600	577	8.2	31.7	47.6	8.4	101	8.2	32.7	50.5	8.3	102	

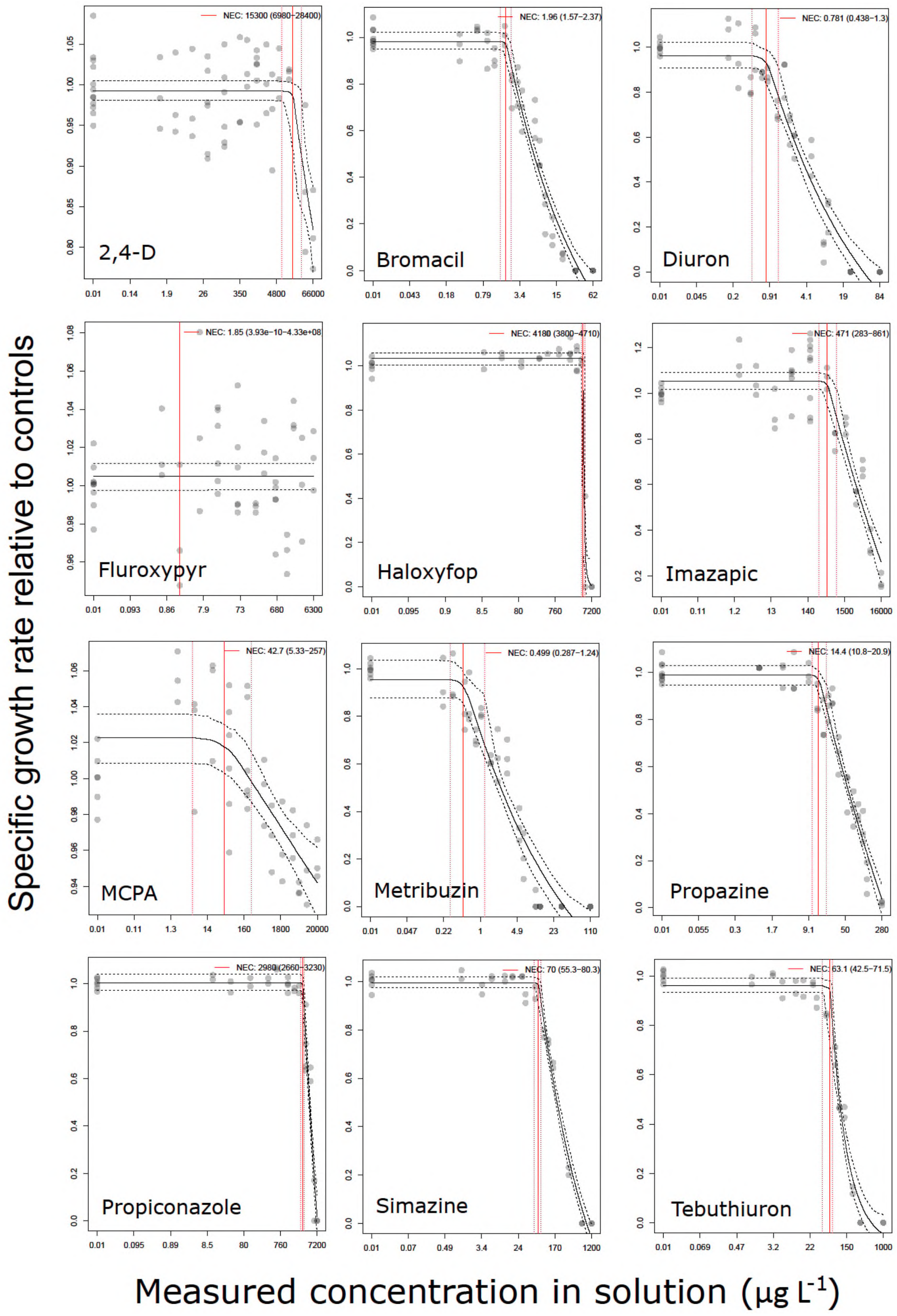

Supplementary Figure 1. Concentration-response curves for NEC derivation. Bayesian non-linear gaussian model fit on the proportional decline in 3-day specific growth rate of *Tisochrysis lutea* sp. relative to the control treatment (solid black line) and Bayesian 95% credible intervals (black dashed line) and the derived no effect concentration (NEC; solid red line) and the 95% confidence interval (red dashed line) of the respective herbicide. All concentrations are in µg L^{-1}. Note the dissimilar scaling on the x-axis.

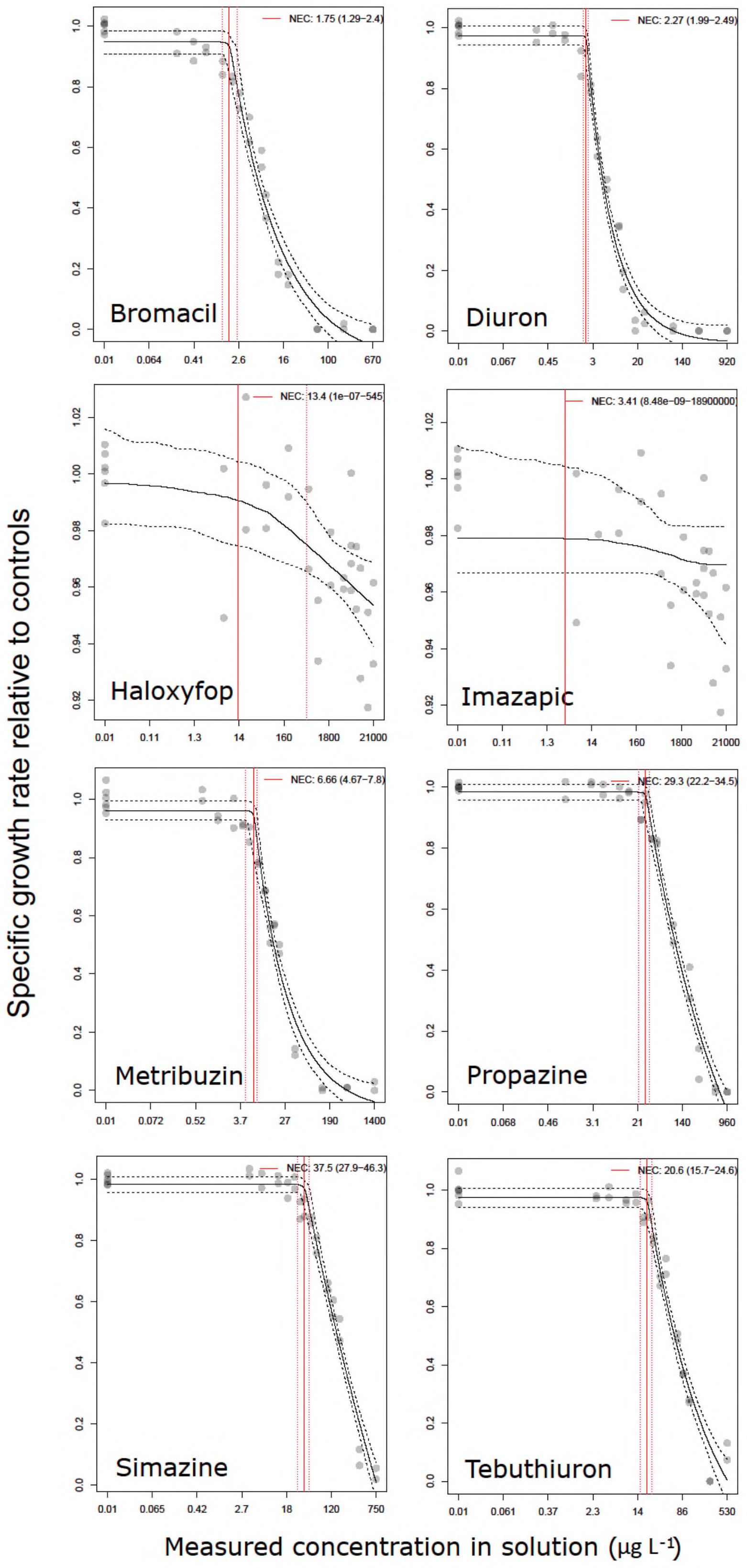

Supplementary Figure 2. Concentration-response curves for NEC derivation. Bayesian non-linear gaussian model fit on the proportional decline in 3-day specific growth rate of *Tetraselmis* sp. relative to the control treatment (solid black line) and Bayesian 95% credible intervals (black dashed line) and the derived no effect concentration (NEC; solid red line) and the 95% confidence interval (red dashed line) of the respective herbicide. All concentrations are in µg L^{-1}. Note the dissimilar scaling on the x-axis.

Supplementary Table 3. Limits of detection (LOD) and recoveries for the analytical method / extract recoveries of grab samples.

Chemical name	Deuterated labelled internal standard compounds	Analytical method LOD (ng mL^{-1})	Method recoveries from water samples ± SD (n=4)
2,4-D	13C6 24D	0.15	86% (± 2.72%)
Bromacil		0.15	
Diuron	Diuron-D6	0.19	51% (± 1.84%)
Fluroxypyr		0.5	
Haloxyfop		0.05	
Imazapic		0.05	
MCPA	MCPA-D6	0.61	85% (± 2.84%)
Metribuzin		0.05	
Propazine		0.5	
Simazine	D10 Simazine	0.13	75% (± 3.31%)
Tebuthiuron		0.13	
